# 

**Published:** 1861-04

**Authors:** 


**NOTICE.**—Let all our readers understand that WE have offered them no inducements whatever to subscribe for the JOURNAL OF HEALTH for the present year; if it can not make its way without people being hired to take it, both people and JOURNAL can go to Guinea or to grass. For one dollar a year we send the JOURNAL—"nothing else."

Lewis's *New Gymnastics*, monthly, $1 a year: Boston. The editor's efforts to establish a rational system of gymnastics merit the patronage and coöperation of all intelligent persons, by his Blow-Gun and Parlor Skates, which he sells at 20 Essex street, Boston. He is enabling the young and the feeble to practice safe, interesting, and highly beneficial modes of exercising and developing the lungs and the limbs.

All subscriptions to the JOURNAL OF HEALTH and FIRESIDE MONTHLY begin with the January number. Any number of the JOURNAL, from the beginning, to complete sets, will be furnished for ten cents. Subscribers who failed to receive their January, February, or March numbers, will have them forwarded without charge.

